# Soybean Inoculated With One Bradyrhizobium Strain Isolated at Elevated [CO_2_] Show an Impaired C and N Metabolism When Grown at Ambient [CO_2_]

**DOI:** 10.3389/fpls.2021.656961

**Published:** 2021-05-20

**Authors:** David Soba, Iker Aranjuelo, Bertrand Gakière, Françoise Gilard, Usue Pérez-López, Amaia Mena-Petite, Alberto Muñoz-Rueda, Maite Lacuesta, Alvaro Sanz-Saez

**Affiliations:** ^1^Instituto de Agrobiotecnología (IdAB), Consejo Superior de Investigaciones Científicas (CSIC)-Gobierno de Navarra, Pamplona, Spain; ^2^Plateforme Métabolisme-Métabolome, Institut de Biologie des Plantes, Université Paris-Sud, Orsay, France; ^3^Department of Plant Biology and Ecology, Faculty of Science and Technology, University of the Basque Country (UPV/EHU), Bilbao, Spain; ^4^Department of Plant Biology and Ecology, Faculty of Pharmacy, University of the Basque Country (UPV/EHU), Vitoria-Gasteiz, Spain; ^5^Department of Crop, Soil, and Environmental Sciences, Auburn University, Auburn, AL, United States

**Keywords:** *Bradyrhizobium* strains, C and N metabolism, elevated [CO_2_], metabolomics, N-fixation, nodule, soybean

## Abstract

Soybean (*Glycine max* L.) future response to elevated [CO_2_] has been shown to differ when inoculated with *B. japonicum* strains isolated at ambient or elevated [CO_2_]. Plants, inoculated with three *Bradyrhizobium* strains isolated at different [CO_2_], were grown in chambers at current and elevated [CO_2_] (400 vs. 700 ppm). Together with nodule and leaf metabolomic profile, characterization of nodule N-fixation and exchange between organs were tested through ^15^N_2_-labeling analysis. Soybeans inoculated with SFJ14-36 strain (isolated at elevated [CO_2_]) showed a strong metabolic imbalance, at nodule and leaf levels when grown at ambient [CO_2_], probably due to an insufficient supply of N by nodules, as shown by ^15^N_2_-labeling. In nodules, due to shortage of photoassimilate, C may be diverted to aspartic acid instead of malate in order to improve the efficiency of the C source sustaining N_2_-fixation. In leaves, photorespiration and respiration were boosted at ambient [CO_2_] in plants inoculated with this strain. Additionally, free phytol, antioxidants, and fatty acid content could be indicate induced senescence due to oxidative stress and lack of nitrogen. Therefore, plants inoculated with *Bradyrhizobium* strain isolated at elevated [CO_2_] may have lost their capacity to form effective symbiosis at ambient [CO_2_] and that was translated at whole plant level through metabolic impairment.

## Introduction

Atmospheric carbon dioxide concentration ([CO_2_]) has increased strongly since preindustrial times (∼280 ppm) to 412.8 ppm registered in November 2020 (^1^ CO2.earth, 2020), and a further substantial increase is expected during this century. Carbon dioxide is the major greenhouse gas of anthropogenic activity origin and has been demonstrated to participate in climate change whereby global temperature and precipitation patterns will be altered (IPCC, 2013). At the plant level, increasing [CO_2_] leads to increased photosynthesis while reducing photorespiration and, as a consequence, increasing growth and seed yield (Ainsworth et al., 2002).

Soybean (*Glycine max* L.) is the fourth most important food crop and the most cultivated legume, with 349 million tons produced in 2018 (FAOSTAT, 2020) being the most traded agricultural commodity, accounting for over 10% of the total value of the global exchange. This legume is a rich source of high-quality proteins and oil and contains a considerable amount of carbohydrates, amino acids, and minerals that contribute to its nutritional value (Medic et al., 2014). Soybean physiological responses to elevated [CO_2_] (e[CO_2_]) have been extensively studied in both controlled and in field environments (Ainsworth et al., 2002; Bishop et al., 2015). Increased photosynthesis due to CO_2_ fertilization leads to increases in leaf carbohydrate content (Ainsworth et al., 2004; Rogers et al., 2006), radiation use efficiency (RUE) (Sanz-Sáez et al., 2017), biomass production, and seed yield (Morgan et al., 2005), while decreasing stomatal conductance (Ainsworth and Rogers, 2007; Soba et al., 2020b) and leaf respiration (Tcherkez et al., 2008).

In some crops, sink limitation and photosynthesis downregulation is sometimes observed in plants grown at e[CO_2_] as a consequence of sugar overaccumulation in the leaves (Ainsworth and Rogers, 2007; Gutiérrez et al., 2009). However, soybeans, as the rest of legume crops, forms symbiotic relationships with *Rhizobiaceae* family bacteria, specifically with *Bradyrhizobium japonicum*, which provide access to atmospheric N_2_, through biological nitrogen fixation (BNF). According to Kaschuk et al. (2009), *Bradyrhizobium* bacteria can consume between 4 and 11% of carbohydrates fixed through photosynthesis and, therefore, increase plant sink capacity and stimulate legume growth under e[CO_2_] avoiding C sink limitation (Ainsworth et al., 2004). On the other hand, BNF and carbohydrate consumption by the nodule is influenced in part by the strain of *Bradyrhizobium* (Kaschuk et al., 2009; Sanz-Sáez et al., 2015). Besides, the microbial population structure in the rizhosphere has been shown to be altered by e[CO_2_] (Wang et al., 2017). Therefore, there is great interest in the isolation and selection for *Bradyrhizobium* strains adapted to future environments, such as e[CO_2_], which hypothetically could be more N_2_-fixation efficient ones compared with unselected or native strains. This hypothesis is reinforced by some studies in alfalfa (Bertrand et al., 2007; Sanz-Sáez et al., 2012) and soybean (Bertrand et al., 2007, 2011; Prévost et al., 2010; Sanz-Sáez et al., 2015) which showed that selected strains could improve legume productivity in response to e[CO_2_] by fixing more N_2_ and hence consuming more C.

Sugawara and Sadowsky (2013) studied different native *B. japonicum* strains from soybean nodules of plants grown and isolated at ambient [CO_2_] (a[CO_2_]) (390 ppm, strain SFJ4-24) and e[CO_2_] (550 ppm, strain SFJ14-36), under fully open-field conditions at a FACE site at the University of Illinois at Urbana-Champaign. They observed that the strain SFJ14-36, isolated at e[CO_2_], significantly overexpressed genes encoding for N_2_-fixation and nodulation in comparison with the strain isolated at a[CO_2_] and the control strain (USDA 110). More recently, Sanz-Sáez et al. (2019) tested if the same strain isolated under e[CO_2_] conditions (SFJ14-36) showed higher BNF and nodule number at e[CO_2_] compared with plants inoculated with USDA110 as it was suggested by the expression profiles published by Sugawara and Sadowsky (2013); however, no statistical differences were observed between plants inoculated with different strains when grown at e[CO_2_]. Therefore, the overexpression of genes observed by Sugawara and Sadowsky (2013) were not matched by a higher BNF. In addition, when the plants were grown at a[CO_2_], those inoculated with the strain isolated at e[CO_2_] showed lower plant fitness in comparison with USDA110. The authors hypothesized that the strains isolated at e[CO_2_] may be attracted only by root exudates produced at e[CO_2_] or that at a[CO_2_] there is some metabolic restrictions in the nodules that reduce the fitness of the symbiotic relationship.

With the objective of better understanding the C and N metabolism interaction in the plants inoculated with different *B. japonicum* strains and grown under e[CO_2_] conditions, that favor yield under climate change conditions, it is needed to study why some rhizobium strains respond better to e[CO_2_] than others, and how this affects plant fitness.

Together and closely related with these physiological responses, plant metabolome is also perturbed under e[CO_2_] (Aranjuelo et al., 2015; Tcherkez et al., 2020). The metabolome of the plant is the link between genotype and phenotype, allowing to study changes in gene expression in response to the environment (Saito and Matsuda, 2008). This makes metabolomic profiling an attractive tool for phenotyping, providing a comprehensive perspective of the environmental changes that influence plants (Obata and Fernie, 2012). In opposition to a traditional metabolic analysis, in which the researcher focuses on a specific class of metabolites related with a metabolic route, the simultaneous profiling of metabolites from biosynthetically unrelated pathways has been demonstrated to increase our understanding of the molecular mechanism that underlies plant response to different stresses (Li et al., 2015). While previous studies have explored the *Rhizobium*-legume specificity and their physiological response to e[CO_2_] (Das et al., 2017; Rabara et al., 2017), to our knowledge, no previous works have analyzed the metabolic profile of soybean nodules and leaves inoculated with different *B. japonicum* strains grown under e[CO_2_] conditions.

The aim of this work was to elucidate the metabolic features involved in *Bradyrhizobium*-soybean specificity under contrasting CO_2_ conditions. With this purpose, metabolic profiling analysis were carried out under two contrasting levels of CO_2_ using the same three *B. japonicum* strains isolated at different [CO_2_] (Sugawara and Sadowsky, 2013) and whose physiologic and photosynthetic parameters were studied by Sanz-Sáez et al. (2019).

## Materials and Methods

### Plant and Bacterial Material

For this study, the same *B. japonicum* strains isolated by Sugawara and Sadowsky (2013) were used: SFJ4-24 (serogroup 123) was a strain isolated from nodules of soybean grown at a[CO_2_] (390 ppm of CO_2_), meanwhile SFJ14-36 (serogroup 38) was a strain isolated from soybean nodules grown at e[CO_2_] (550 ppm of CO_2_). As a control, we used USDA110 strain because it has demonstrated high soybean performance at ambient and elevated [CO_2_] at SoyFACE facility at the University of Illinois at Urbana-Champaign^2^ (Sanz-Sáez et al., 2015). Soybean cultivar (*Glycine max* cv. 93B15; Pioneer Hi-Bred) was used in the same way as being used by Sugawara and Sadowsky (2013) in order to avoid problems of compatibility between the soybean cultivar and the *Bradyrhizobium japonicum* strains. These strains were provided by Prof. Michael Sadowsky (University of Minnesota; SFJ4-24, and SFJ14-36) and by USDA-ARS Rhizobium Germplasm Resource Collection in Belstville, MD (USDA110). The different *B. japonicum* cultures were made exactly as explained in Sanz-Sáez et al. (2019).

### Plant Growth Conditions, Treatments, and Sampling

Soybean seeds were surface sterilized with sodium hypochlorite (1%) for 10 min and rinsed with sterile water until the smell of bleach disappeared. For the seed inoculation, 200 ml of medium liquid culture containing ≈5 × 10^9^ cell ml^–1^ of each individual *B. japonicum* strain was centrifuged for 15 min at 5,100 × *g* to separate the bacteria from the media. The pellet containing the bacteria was resuspended in 2 ml of sterile deionized water containing 2% polivinylpolypyrrolidone (PVPP) reaching a concentration of ≈10^11^ cel ml^–1^. Then 200 seeds were placed in a 500-ml sterile beaker containing 2 ml of concentrated inoculum on a rotatory shaker overnight, and later immediately planted. For the liquid inoculation, 1 L of liquid culture containing ≈5 × 10^9^ cell ml^–1^ was centrifuged and resuspended as above to a final concentration of 10^8^ cell ml^–1^.

Five inoculated soybean seeds were planted in 10 L pots containing a mixture of peat moss, perlite, and vermiculite of 1:1:1 *v*/*v*/*v* that was previously sterilized as described in Sanz-Sáez et al. (2015). One week after emergence, plants were thinned to one plant. After plants emerged from the pot, they were inoculated three times coinciding with 2, 9, and 16 days after emergence (DAE) with the liquid inoculum from each *B. japonicum* strain (USDA110, SFJ4-24, and SFJ14-36). All plants were watered alternatively with Evans N-free solution and distilled water to avoid salt accumulation (Moore, 1974). Soybean plants were grown in two growth chambers (Phytotron Service, SGIker) at the University of the Basque Country (UPV/EHU), from the beginning of the experiment, one maintained at a[CO_2_] (≈400 ppm CO_2_), and other maintained at e[CO_2_] (700 ppm CO_2_). Both chambers were maintained at 60/70% day/night relative humidity and 25/22°C day/night temperature, and a photosynthetic photon flux density (PPFD) of 1,200 μmol m^–2^ s^–1^ from 7:00 to 22:00 h, until developmental stage V5 (Fehr et al., 1971), when the day length was decreased by 2 h to induce flowering. Every 2 weeks, plants and CO_2_ treatments were rotated among and within chambers in order to reduce potential chamber effects. Harvest was carried out when plants reached full flowering developmental stage (R2). This stage was selected because flowering is the period when N_2_ fixation is supposed to be in its peak, and nodules have not started to senesce (Rogers et al., 2006). At this moment, six plants per inoculation and CO_2_ treatment were harvested and separated into organ samples; leaves, stems, roots, and nodules. Each organ sample was oven dried at 65°C for at least 72 h and then weighed. The data is presented as weight of each organ separately (g of dry weight plant^–1^), and stacked by total dry weight. For metabolic analysis, samples of leaves and nodules from three plants were collected and immediately frozen in liquid nitrogen and stored at −80°C until analysis.

### Nitrogen Isotopic Composition Analyses

Twenty-four hours before harvest, the underground zone was enriched with ^15^N_2_, and after harvest, the labeled ^15^N incorporation [measured as stable ^15^N isotope composition (δ^15^N)] in nodule tissue was measured. This parameter has been recently used as a measure of nodule performance in soybean and other legumes (Soba et al., 2020a) showing that the higher the nodule δ^15^N the greater the BNF. Three plants (there was one plant per pot) per inoculation and CO_2_ treatment were labeled, while three plants were used as unlabeled controls and harvested at the same time as the labeled plants. The ^15^N_2_ labeling was accomplished by injecting labeled gas into the root zone using handmade labeling pots following the procedure of Sanz-Sáez et al. (2015). Plants were grown in these pots for the duration of the experiment. On the night preceding the labeling experiment, the pots were sealed with plastic lids in order to avoid the escape of the labeled gas. To perform the ^15^N_2_ labeling, 10% ^15^N_2_-enriched gas was prepared in Supelco-Inert Foil Gas Sampling Bags (Sigma-Aldrich, St Louis, MO, United States) by mixing the ^15^N_2_-labeled gas enriched at 99% with ambient air (δ^15^N_2_ at 0‰). Two hundred milliliters of ^15^N_2_ (10%) mixed gas was injected into the labeling pots using a gas syringe (SGE; Sigma-Aldrich) 2 and 4 h after the lights were turned on, coinciding with the period of greatest N_2_-fixing activity (Molero et al., 2019). After 24 h, the plants were harvested and separated into nodules, roots, and leaves, then dried at 65°C for at least 72 h. The dried organs were weighed and ground to 1 mm particle size. The samples were analyzed in a Costech 4010 elemental analyzer coupled in continuous flow with a Thermo Fisher Delta V Advantage Isotope Ratio Mass Spectrometer (IRMS Thermo Scientific, Waltham, MA, United States). The ^15^N/^14^N ratio in soybean nodule, root, and leaf material was expressed in δ notation (δ^15^N) following the equation as described in Sanz-Sáez et al. (2019). The amount of ^15^N fixed during the day of the labeling experiment was calculated as ^15^N fixed in each organ following the equation, as described in Bei et al. (2013).

### Estimation of Rate of RuBP Oxygenation From Gas Exchange Measurements

Photorespiratory rates were estimated as the rate of ribulose-1,5-bisphosphate (RuBP) oxygenation (*v*_*o*_) derived from the measured rates of CO_2_ uptake by the leaves according to Sharkey (1988) and von Caemmerer (2000), as described in Noctor et al. (2002):

v=o(A-R)d/(1/O:C-0.5),

where *A* is the measured rate of net CO_2_ uptake, *R*_d_ is non-photorespiratory CO_2_ release in the light (taken as 50% of the rate measured in the dark in each experiment) and *O:C* is the ratio of RuBP oxygenation to carboxylation. The ratio *O:C* was calculated as:

O:C=(1/S)rel(O/cC)c

where *S*_rel_ is the specificity factor of Rubisco (taken as 110; Keys, 1999), and *C*_c_ and *O*_c_ are the chloroplastic concentrations of CO_2_ and O_2_, respectively. *O*_c_ was assumed to be that of water in equilibrium with air at 20°C (276 μM) and *C*_*c*_ was derived from *C*_*i*_ by taking a CO_2_ transfer conductance through the mesophyll (*g*_*i*_) of 0⋅32 mol m^–2^ s^–1^ (Gillon and Yakir, 2000) and assuming that the rate of CO_2_ uptake affects *C*_*c*_ relative to *C*_*i*_ as in Ruuska et al. (2000):

C=cC-iA/gi

where *C*_*c*_ was converted to a molar concentration by applying a CO_2_ solubility constant at 20°C of 0.0392 mol L^–1^ (von Caemmerer, 2000). The gas exchange parameters *A*, *R* in the dark, and *C*_*c*_ were measured exactly as described in Sanz-Sáez et al. (2019).

### Metabolic Analyses

Leaf and nodule samples (20 mg of powder from freeze-dried material) were ground in a mortar in liquid nitrogen, and then in 2 ml of 80% methanol, in which ribitol (100 μmol L^–1^) was added as an internal standard. After centrifugation at 15,000 rpm for 15 min at 4°C, the supernatant was collected and centrifuged again. Then, the supernatants were spin-dried under vacuum and stored at −80°C until analysis. Relative metabolite content was determined by gas chromatography coupled to time-of-flight mass spectrometry (GC-MS) using a LECO Pegasus III coupled to an Agilent 6890N GC system. Sample derivatization and GC-MS analyses were carried out as described in Aranjuelo et al. (2015). Peak identity was established by comparison of the fragmentation pattern with MS-available databases (NIST). The integration of peaks was performed using the LECO Pegasus software and the automated peak integration was verified manually for each compound in all analyses. The quantification was normalized to dry weight (DW) to avoid any discrepancy due to changes in relative water content.

### Statistical Analysis

Statistical analyses were performed with IBM SPSS Statistics for Windows, Version 20.0 (IBM Co., Armonk, NY, United States). Differences among the three *Bradyrhizobium* strains and the two CO_2_ levels were evaluated by two-way analyses of variance (ANOVA), with the strain and CO_2_ as fixed factors. All data were tested for normality (Kolmogorov–Smirnov test) and homogeneity of variances (Levene’s test). For ANOVA analysis, the results were considered to be significant when *p* < 0.05. In order to reduce the multivariate data complexity and identify patterns between samples, principal component analysis (PCA) was performed for nodules and leaves and the 121 metabolites were taken into account. Heat map and PCA for the two organs were conducted using XLSTAT 2008 (Addinsoft, Paris, France) software. Heat maps were done independently for leaves and nodules with metabolites showing significant differences between *B. japonicum* strain, [CO_2_], and/or interactions in each tissue. Clustering was based on the Pearson’s correlation coefficients among the metabolites. In this manuscript, the red color is proportional to a lower concentration; conversely, the intensity of the green color is proportional to higher concentration values.

## Results

### Biomass and Physiologic Measurements

**FIGURE 1:**
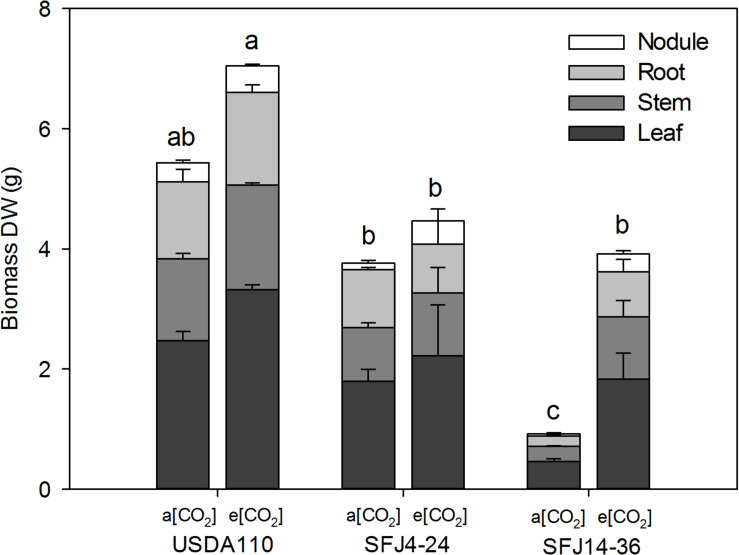
Effect of [CO_2_] on soybean biomass in plants inoculated with three different *B. japonicum* strains. Nodule, root, stem, and leaf biomass (g DW plant^– 1^) of soybean plant grown at a[CO_2_] (400 ppm) and e[CO_2_] (700 ppm) and inoculated with three *Bradyrhizobium japonicum* strains (USDA110, SFJ4-24, and SFJ14-36). Bars correspond to the mean ± SE of *n* = 6 of the biomass of each tissue. Results of statistics for total biomass (the sum of nodule, root, leaf, and stem) are shown (two-way ANOVA, *P* < 0.05). Different letters indicate significant differences (Tukey *post hoc* test *P* < 0.05).

At the R2 stage, strain, CO_2_ level, and their interaction were found to have a significant effect on biomass of leaves, stems, and roots (data not shown). In contrast, nodule weight was only significantly affected by CO_2_ level. Elevated [CO_2_] significantly increased total biomass in all studied organs for the plants inoculated with *B. japonicum* strain isolated at e[CO_2_] (SFJ14-36) but not for the reference strain (USDA110) or in the strain isolated at a[CO_2_] (SFJ4-24) (Figure 1). However, the final value at e[CO_2_] of SFJ14-36 strain was similar to the SFJ4-24 strain. Plants inoculated with USDA110, the reference strain, showed the highest values at both CO_2_ concentrations (Figure 1).

**FIGURE 2:**
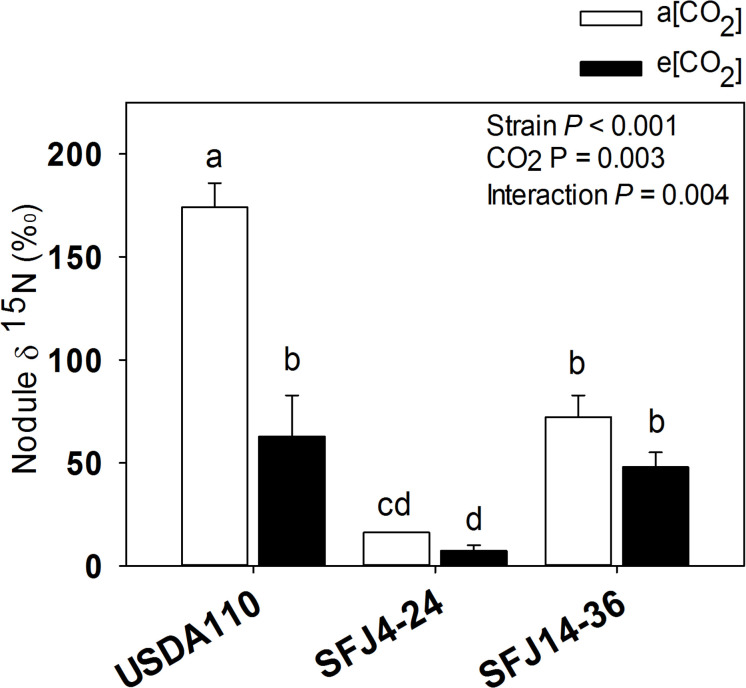
Effect of [CO_2_] on soybean nodule δ^15^N in plants inoculated with three different *B. japonicum* strains. Nodule ^15^N isotope labeling (δ^15^N, ‰) ± of soybean plants grown at a[CO_2_] (400 ppm) and e[CO_2_] (700 ppm) and inoculated with three *Bradyrhizobium japonicum* strains (USDA110, SFJ4-24, and SFJ14-36). Bars correspond to the mean ± SE of *n* = 3. Results of statistics are shown (two-way ANOVA, *P* < 0.05). Different letters indicate significant differences (Tukey *post hoc* test *P* < 0.05).

**FIGURE 3:**
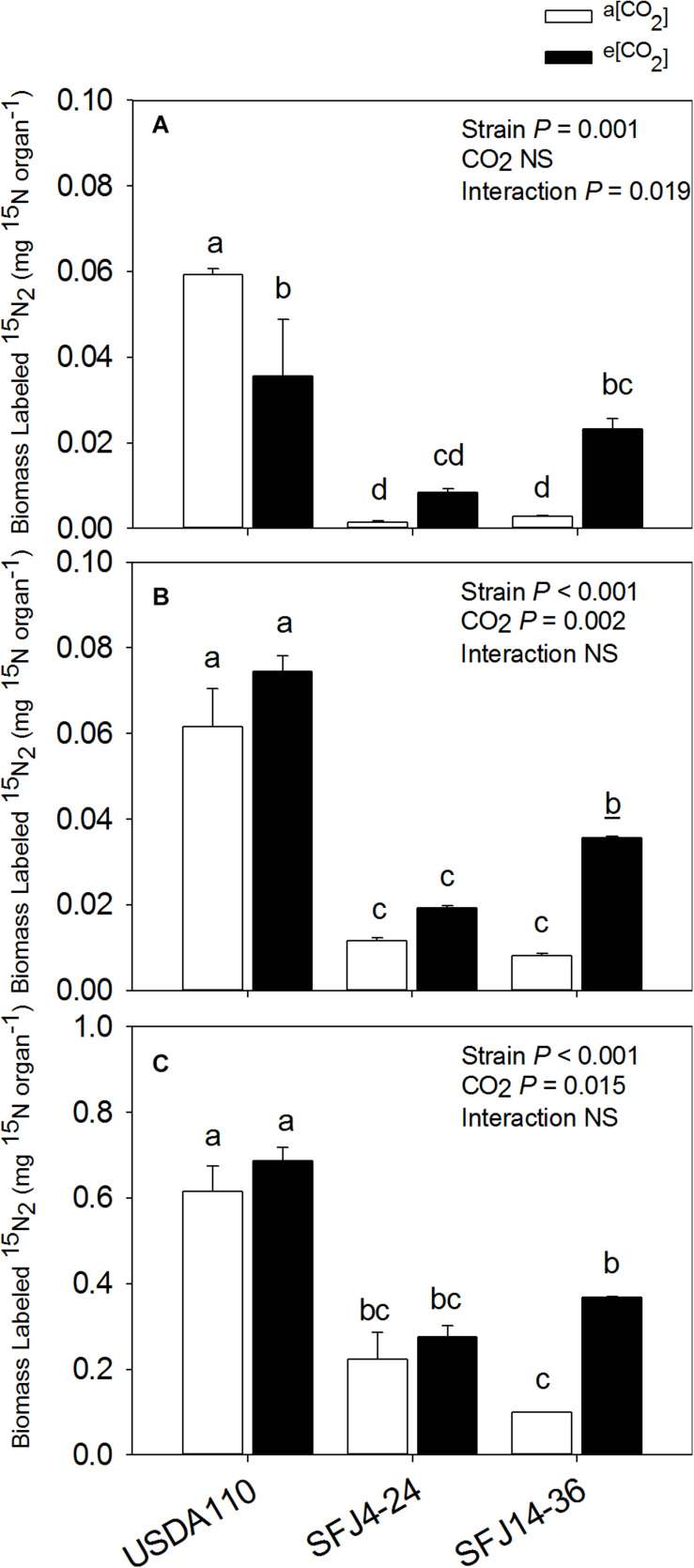
Effect of [CO_2_] on biomass labeled ^15^N in three organs of soybean plants inoculated with three different *B. japonicum* strains. Biomass labeled ^15^N (mg ^15^N organ^– 1^) in **(A)** nodules, **(B)** roots, and **(C)** leaves of soybean grown at a[CO_2_] (400 ppm) and e[CO_2_] (700 ppm) and inoculated with three *Bradyrhizobium japonicum* strains (USDA110, SFJ4-24, and SFJ14-36). Bars correspond to the mean ± SE of *n* = 3. Results of statistics are shown (two-way ANOVA, *P* < 0.05). Different letters indicate significant differences (Tukey *post hoc* test *P* < 0.05).

For nodule ^15^N isotope labeling (δ^15^N), greatest value was found in nodules of plants inoculated with USDA110 strain grown at a[CO_2_] which was significantly higher than plants grown at e[CO_2_] (Figure 2). Interestingly, nodules of SFJ4-24 and SFJ14-36 did not show significant differences between CO_2_ levels, and the δ^15^N was significantly greater in nodules of SFJ14-36 when compared with SFJ4-24 (Figure 2). For a more holistic vision, we calculated the amount of δ^15^N per organ (mg ^15^N organ^–1^) (Figure 3). The results showed a clear significant effect of CO_2_ for strain SFJ14-36 in the three studied tissues (leaf, root, and nodule) in contrast with USDA110 and SFJ4-24 strains.

**FIGURE 4:**
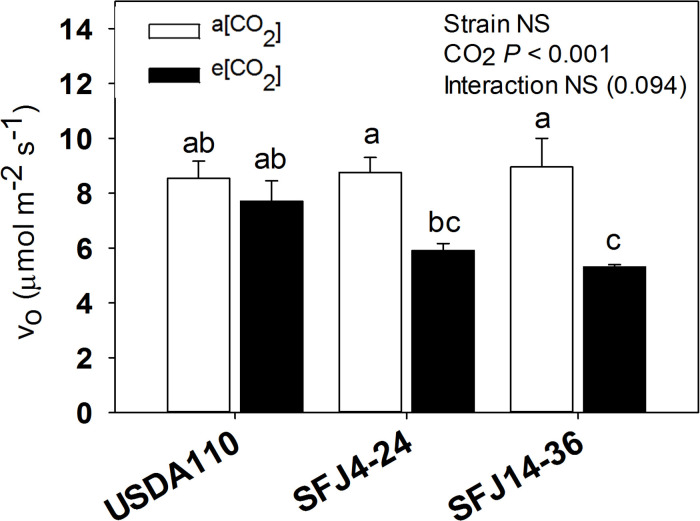
Photorespiratory estimations from gas exchange measures. Estimated rate of RuBP Oxygenation (*v*_o_) in soybean leaves grown at a[CO_2_] (400 ppm) and e[CO_2_] (700 ppm) and inoculated with three *Bradyrhizobium japonicum* strains (USDA110, SFJ4-24, and SFJ14-36). Bars correspond to the mean ± SE of *n* = 6. Results of statistics are shown (two-way ANOVA, *P* < 0.05). Different letters indicate significant differences (Tukey *post hoc* test *P* < 0.05).

The estimated rate of photorespiration (*v*_o_) from gas exchange measures showed significant differences between CO_2_ levels. Whereas both SFJ4-24 and SFJ14-36 showed a significant decrease of photorespiration under e[CO_2_], USDA 110 did not (Figure 4).

### Metabolite Patterns

**FIGURE 5:**
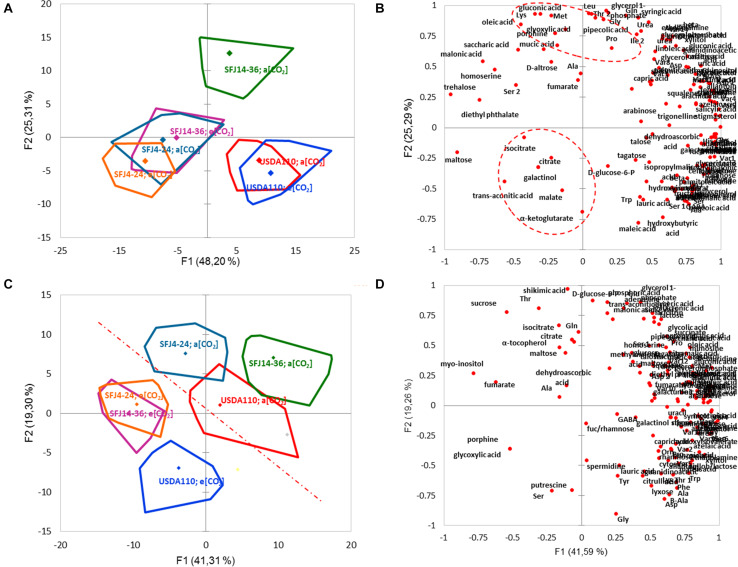
Graphical representation of metabolomic response to different [CO_2_] in soybean plants inoculated with three *B. japonicum* strains. Principal component analysis (PCA) [**(A)** nodules and **(C)** leaves] of the different metabolites in soybean plants inoculated with three *B. japonicum* strains. Scatter plot distribution [**(B)** nodules and **(D)** leaves] of the different metabolites in soybean plants inoculated with three *B. japonicum* strains. Three replicates were examined per strain and CO_2_ level.

**FIGURE 6:**
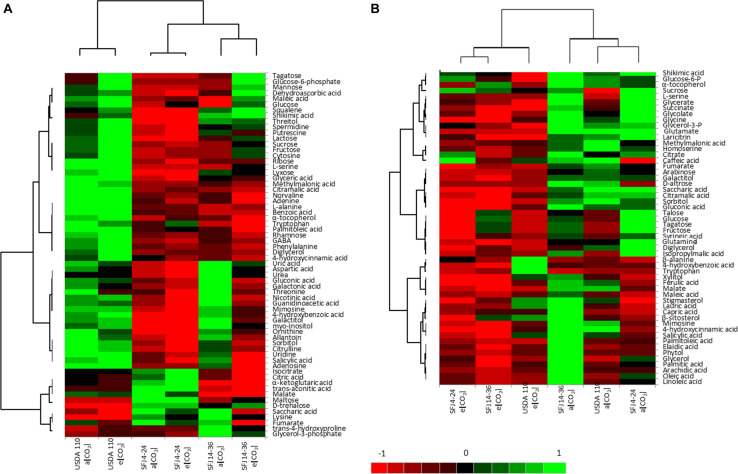
Differentially expressed metabolites involved in [CO_2_] responses in function of *B. japonicum* strain. **(A)** Hierarchically clustered heat maps of metabolites that were found to be significantly different between *Bradyrhizobium* strains, [CO_2_], and their interaction in nodules. **(B)** Hierarchically clustered heat maps of metabolites that were found to be significantly different between *Bradyrhizobium* strains, [CO_2_], and their interaction in leaves. Each column represents the average of three replicates. Intensity of red and green indicates increases and decreases relative to the mean respectively, as shown in the color scale.

Metabolite profiling was performed by gas chromatography coupled with time-of-flight mass spectrometry (GC-MS), and 121 different metabolites were identified by reference to their MS data. These metabolites were classified in eight chemical groups (organic acids, amino acids, sugars, fatty acids, polyols, nucleotides, secondary metabolites, and others), and their relative abundance is shown in Figure S1. The statistical analysis (PCA and heat map hierarchical clustering) of leaf and nodule metabolomic profile indicates that a clear differentiation between organs could be done (Figures 5, 6). While in the nodule metabolome, *B. japonicum* strain was found to have the main effect, with little impact of CO_2_ level (except for SFJ14-36) (Figure 5A), in leaf metabolome, CO_2_ level had the main effect (Figure 5C). Nodule PCA analysis revealed that the two principal components explained 73.5% of total variation between strains and CO_2_ levels (Figure 5A). The six combinations of strains and CO_2_ levels were grouped by strains without differences between treatments except for the SFJ14-36, which showed significant differences between CO_2_ levels. The loading plot revealed that the discrimination of samples by PC1 (48.2% of the total variance) was, in part due to sugars such as maltose and trehalose, whereas amino acids like lysine, methionine, threonine, leucine, glycine, and glutamine contributed to the separation of samples by PC2 (25.3% of the total variance). It is interesting to note that Krebs cycle related metabolites are grouped in the lower-left quadrant of the loading plot (Figure 5B). On the other hand, leaf PCA analysis showed a more unrelated distribution of strains and treatments, but a clear separation between *Bradyrhizobium* strains grown at ambient and elevated [CO_2_] could be traced (Figure 5C). The two principal components explained 60.6% of total variation between strains and CO_2_ levels. No distinctive pattern in metabolites were detected to explain this variation; nevertheless, important metabolites such as D-glucose-6-phosphate, glycine, serine, the polyamines putrescine and spermidine, and shikimic acid were important contributors to the separation of samples by PC2 (Figure 5D).

Of the 121 analyzed metabolites, 64 were significantly affected by CO_2_, strain or their interaction in nodules and 54 in leaves (Figure 6). In order to provide a better understanding of this variation, metabolite profiling representation (heat map) and hierarchical clustering analysis was undertaken separately for each organ (Figure 6). In nodules, the hierarchical clustering of the six combinations (two levels of CO_2_ and three *Bradyrhizobium* strains) formed three clusters, one for each strain with little differences between CO_2_ treatments, except for the strain SFJ14-36 where differences between CO_2_ levels were bigger (Figure 6A), as we have seen in the PCA analysis (Figure 5A). On the other hand, the hierarchical clustering of the 64 significant metabolites allowed the grouping of metabolites into two major clusters. Cluster 1 was mostly made of metabolites in higher concentration in plants inoculated with USDA110 strain as compared with other strains and could be subdivided in two subclusters; 1A: sugars (glycolysis pathway: sucrose, glucose, fructose, mannose, etc.) and 1B: N-related compounds such as ureides and urea cycle metabolites (allantoin, uric acid, ornithine, aspartic acid, citrulline, urea, etc.). On the contrary, cluster 2 was formed of metabolites in lower concentration in USDA110 strain and mostly included Krebs cycle metabolites (fumarate, malate, citrate, isocitrate, α-ketoglutarate, etc.) (Figure 6A).

**FIGURE 7:**
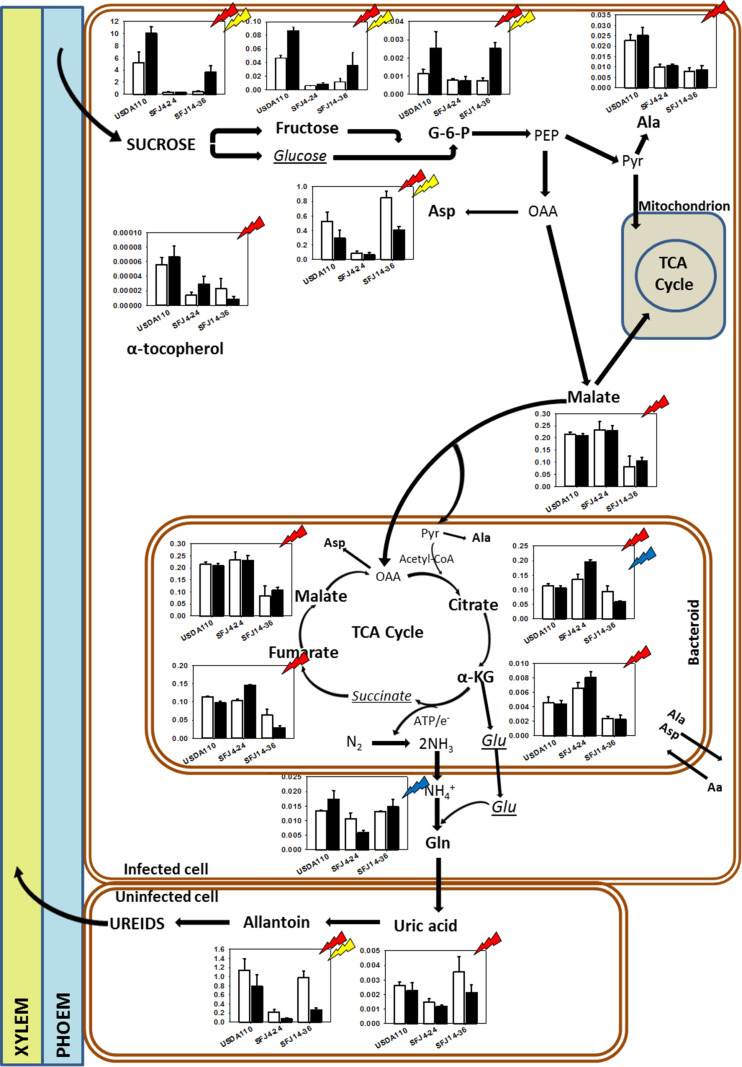
Effects of *Bradyrhizobium japonicum* strain and [CO_2_] on soybean nodule metabolism. Bar charts showing the relative abundance of metabolites in ambient [CO_2_] (white) and elevated [CO_2_] (black) in soybean plants inoculated with three different *Bradyrhizobium japonicum* strains. Red, yellow, and blue lightning signs indicate the significant effect of strain, CO_2_, and their interaction, respectively. Metabolites in bold indicate metabolites that were found to have significant effect of strain, CO_2_, and/or their interaction; italic metabolites indicate metabolites analyzed that were not found to have significant effect of strain, CO_2_, and their interaction, and not bold neither italic indicates metabolites not analyzed.

**FIGURE 8:**
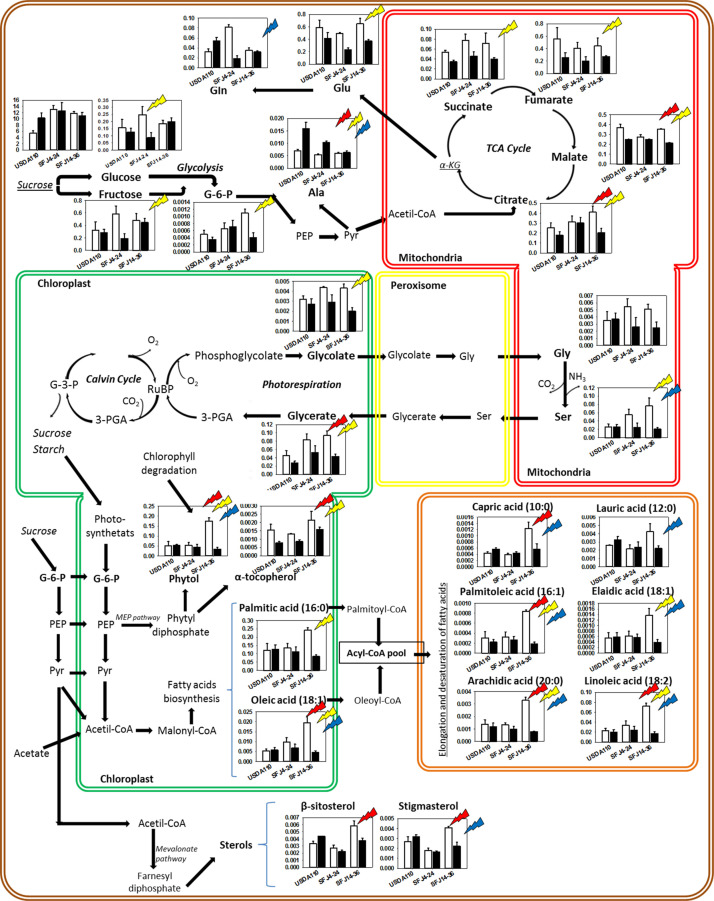
Effects of *Bradyrhizobium japonicum* strain and [CO_2_] on soybean leaf metabolism. Bar charts showing the relative abundance of metabolites in ambient [CO_2_] (white) and elevated [CO_2_] (black) in soybean plants inoculated with three different *Bradyrhizobium japonicum* strains. Red, yellow, and blue lightning signs indicate the significant effect of strain, CO_2_, and their interaction, respectively. Bold metabolites indicate metabolites analyzed that were found to have significant effect of strain, CO_2_ and/or their interaction; italic metabolites indicate metabolites analyzed that were not found to have significant effect of strain, CO_2_, and their interaction, and not bold neither italic indicates metabolites not analyzed.

Contrary to nodules, in leaves, the hierarchical clustering of CO_2_ levels and *Bradyrhizobium* strain combinations was grouped by CO_2_ level in two clusters (Figure 6B). Cluster 1 formed by plants grown at e[CO_2_] and cluster 2 by plants grown at a[CO_2_]. In general, the metabolites were accumulated in greater quantity at a[CO_2_]. The hierarchical clustering of the 54 significant metabolites showed also two different clusters. Cluster 1 was formed by two subclusters mainly composed by metabolites related with photorespiration (glycolate, glycine, serine, glycerate) and Krebs cycle (glutamate, citrate, succinate, etc.) and sugar metabolites (fructose, glucose, tagatose, talose, sorbitol, etc.). On the other hand, cluster 2 showed a greater accumulation of metabolites in plants inoculated with SFJ14-36 and lower in SFJ4-24 strain at a[CO_2_]. This cluster was mainly composed of lipid-related metabolites (free fatty acids and sterols). Figures 7, 8 represent metabolites that were significantly affected by *B. japonicum* strain, [CO_2_], or their interaction either on soybean nodule and leaf.

## Discussion

### Physiologic Parameters

Our study showed that [CO_2_] effect on soybean biomass was tightly dependent on the *B. japonicum* strain analyzed. While in SFJ4-24 and USDA110 changes between CO_2_ levels were not significant, soybeans inoculated with SFJ14-36 strain showed an increase of 322% in their total biomass under e[CO_2_] (Figure 1). Both, N_2_ fixation and photosynthetic performance were involved in such different responses. When we analyze δ^15^N in nodules of SFJ14-36, used as a measure of BNF-specific rate, no significant differences were found. However, significant differences, in nodule, root, and leaf, were found when the amount of total biomass labeled ^15^N_2_ in this strain was calculated relating with the total N fixed by BNF and their translocation to other tissues (Figures 2, 3). The greater ^15^N content under e[CO_2_] in SFJ14-36 compared with a[CO_2_] was in contrast with the lack of significant differences between CO_2_ level for the other two strains in roots and leaves. This difference may be due to a better translocation of fixed N to the aboveground tissues as shown by nodule metabolite data (greater accumulation of aspartic acid (Asp) and allantoin in nodules of plants inoculated with SFJ14-36 at a[CO_2_]) (Figure 7). Briefly, in the plants inoculated with the strain isolated at e[CO_2_] (SFJ14-36), even with a similar BNF-specific rate at both CO_2_ levels, the greater nodule biomass at e[CO_2_] leads to a greater amount of fixed N and this together with a better translocation to the aerial parts, allows a better biomass growth under e[CO_2_]. However, nodulation in soybean that were inoculated with SFJ14-36 strain seems to be restricted or delayed when grown at a[CO_2_] as previously showed by Sanz-Sáez et al. (2019) compromising the whole plant fitness due to a lower N availability. None of these facts seems to occur in the model strain (USDA110) or in the one isolated at a[CO_2_] (SFJ4-24).

In addition to BNF, gas exchange measures also contributed to explain the different responses of plants inoculated with different *B. japonicum* strains to e[CO_2_]. As previously showed by Sanz-Sáez et al. (2019), photosynthesis was reduced when plants grew at a[CO_2_], but this reduction was significantly greater in SFJ14-36 strain when compared with the other two. One of the most important parameters affecting C fixation through photosynthesis is photorespiration. Zhu et al. (2008) estimated a reduction of gross C3 photosynthesis efficiency by 48% at current [CO_2_] and temperature conditions, mainly associated with the consumption of fixed C and energy in the glycolate recycling process. As observed in Figure 4, both SFJ4-24 and SFJ14-36 reduced the estimated *v*_o_ (32.5 and 40.7%, respectively) when grown at e[CO_2_]; in contrast, values in USDA110 did not change. Therefore, in SFJ4-24 and SFJ14-36, the increase in the photosynthetic rate previously observed under e[CO_2_] could be in part due to a reduction in the photorespiratory rate, especially in SFJ14-36 as validated below with the metabolic data that shows a decrease in intermediates of the glycolate cycle (glycolic acid, glycine, and serine among others) (Figure 6) in leaves grown under e[CO_2_].

The decreased nodulation and reduction of the symbiotic fitness of the plants inoculated with SFJ-14-36 and grown at a[CO_2_] could be due to changes in the quantity and/or quality of phenolic substances excreted by the roots (Sugawara and Sadowsky, 2013; Wang et al., 2017). At e[CO_2_], roots excrete more and different phenolic compounds that attract rhizobium species (Sugawara and Sadowsky, 2013). As SFJ-14-36 was isolated under e[CO_2_], an effective nodulation may have been dependent of phenolic substances only emitted under these circumstances whereas at a[CO_2_], these compounds may have changed (Sugawara and Sadowsky, 2013), reducing the nodule formation and the amount of N that was fixed and fed to the plant. Another reason for the lower nodulation of plants inoculated with SFJ14-36 strain at ambient [CO_2_] could be the lack of synergy between *Bradyrhizobium* strain and soybean cultivar. This is not likely because this study used the same soybean cultivar (cv. 93B15; Pioneer Hi-Bred) used in Sugawara and Sadowsky (2013) when the strain was isolated in e[CO_2_] at the SoyFACE facility in Illinois.

Together with physiologic parameters, plant metabolomic can give us valuable information about the status of the soybean-*Bradyrhizobia* symbiosis under current and future [CO_2_]. As shown, [CO_2_] effect on physiology was dependent on the *B. japonicum* strain and these differential responses are expected to be reflected in the accumulation of specific metabolites. Therefore, metabolomics are a valuable tool to decode the physiologic differences between plants inoculated with different strains and CO_2_ levels, helping us understand why the plants symbiosis with the strain that was isolated at e[CO_2_] does not perform well at a[CO_2_] or why USDA110 is more effective than the other two strains at a[CO_2_].

### Carbon and Nitrogen Metabolism

Nitrogen fixation in legume nodules is fueled by C fixed through photosynthesis. The current experiment showed that the amount of sucrose in nodules was affected by CO_2_ level and by *Bradyrhizobium* strain. Plants inoculated with USDA110 showed the highest sucrose levels (especially at e[CO_2_]); on the contrary, SFJ4-24 showed almost no sucrose in both ambient and elevated [CO_2_] when compared with USDA110 (Figure 7). This strain-specific sucrose content was in opposition with the lack of statistically photosynthetic differences observed by Sanz-Sáez et al. (2019) between USDA110 and SFJ4-24. Such results point to the fact that other reasons may exist behind this contrasting sucrose content between plants inoculated with the two strains. More specifically, obtained data would reveal that a greater use of sucrose in the leaf as observed in metabolites implied in glycolysis (glucose, fructose) in leaves of SFJ4-24 at a[CO_2_] (Figure 8) which is in accordance of the higher respiration rates observed in leaves of plants inoculated with this strain (Sanz-Sáez et al., 2019) which could limit its export to nodules. On the other hand, nodules of plants inoculated with SFJ14-36 showed greater amount of sucrose under e[CO_2_] than under a[CO_2_] probably due to a better photosynthetic rate at e[CO_2_]. Also, metabolites involved in glycolysis (fructose, glucose, and glucose-6-phosphate) were significant affected by both, *Bradyrhizobium* strain and CO_2_ level (Figure 7). These observations suggest a poor supply and/or rapid consumption of C for respiration and C skeletons in nodules of plants inoculated with SFJ4-24 and SFJ14-36 under a[CO_2_] (Aranjuelo et al., 2014). Surprisingly, when we observed the content of malate, the main dicarboxylic acid formed after glycolysis (Lodwig and Poole, 2003), and other organic acids involved in Krebs cycle, we saw that they were affected by *Bradyrhizobium* strain but not by CO_2_ level content. On the other hand, malate content in SFJ4-24 nodules was similar to USDA110 and higher than in SFJ14-36. These two observations could suggest that in nodules of plants inoculated with SFJ4-24, much of the carbon glycolyzed was derived to maintain nodule energy supply through malate production. Meanwhile in USDA110, although malate content is similar to that observed in SFJ4-24, the preceding substrates (sucrose, fructose, and G-6-P) were at much higher concentrations indicating that part of this C was diverted to the production of other compounds, such as phenolic compounds that were in higher concentration than in the other strains (Figure 6), suggesting an active carbon metabolism in soybeans inoculated with USDA110. This depletion in Krebs intermediates could be replenished trough the GABA-shunt allowing the synthesis of succinate that can enter the Krebs cycle (Saiz-Fernández et al., 2020) maintaining high its activity and the levels of malate observed with this strain. This role of the GABA-shunt would be confirmed by the increase of the levels of GABA and polyamines (spermidine and spermine) observed in USDA110 under e[CO_2_] (Figure 6) as it was proposed by Saiz-Fernández et al. (2020) in corn plants. The low malate content in plants inoculated with SFJ14-36 was also showing this C diversion in plants infected by this strain. A significant portion of carbon entering in the Krebs cycle in bacteroids is diverted, *via* anaplerotic reactions, into some amino acids: alanine (Ala), through pyruvate (Igamberdiev and Kleczkowski, 2018); aspartic acid (Asp), through oxaloacetate (OAA) (Melzer and O’Leary, 1991); and glutamic acid (Glu), through α-ketoglutarate (α-KG) (Salminen and Streeter, 1987, 1992; Figure 7).

Aspartic acid and Glu were by far the two most abundant amino acids in nodules; whereas by contrast, the content of Ala was low (Figure 7). Aspartic acid is a common amino acid and, in nodulated soybean, has been shown to be a form of N-transport, especially under N-stress conditions (Lima and Sodek, 2003). In our work, the highest content of Asp was found in nodules of plants inoculated with SFJ14-36 at a[CO_2_], in special when compared with SFJ4-24 strain. However, if a great part of OAA is transaminated to Asp, the Krebs cycle will be shut down because malate and citrate cannot be synthesized (Hayes, 2001), explaining the low content of Krebs cycle metabolites observed in SFJ14-36 strain. Therefore, in nodules, the observed differences in *Bradyrhizobium* strains between glycolysis metabolites and organic acids involved in Krebs cycle may be due to a diversion of C to Asp production *via* anaplerotic reaction; and as a result, lesser organic acids were involved in energy production for N_2_ fixation in plants inoculated with SFJ14-36 strain. In the case of USDA 110, the diversion of C from Krebs cycle to phenolic compounds could be replenished through the GABA-shunt.

Although soybean is a ureides exporter, previous studies have revealed that plants with impaired N_2_ fixation have shown an enhancement of Asp transport through xylem sap, as a precursor of the products of NH_4_^+^ assimilation (Puiatti and Sodek, 1999; Lima and Sodek, 2003). Additionally, when photoassimilates transport to nodules is restricted, as it seems to be the case in plants inoculated with SFJ14-36 strain grown at a[CO_2_], plants may use C, N, and energy in a more efficient way, through the carboxylation of Phosphoenolpyruvate (PEP) into OAA, required for Asp synthesis, instead entering Krebs cycle through malate (Fischinger and Schulze, 2010). This mechanism has been shown mainly in indeterminate nodules (pea); however, Silvente et al. (2003) showed that in ureides exporter nodules (bean), aspartate aminotransferase (AAT) may be acting as an important switching enzyme in driving the metabolic flow of fixed N through amide or ureides synthesis, helping to explain the significant higher concentration of Asp and Krebs cycle metabolites in nodules of plants inoculated with SFJ14-36 strain at a[CO_2_] (Figure 7). The use of Asp for N assimilation is connected with the carboxylation of PEP, while any use of C through Krebs cycle for N assimilation is connected with a preceding decarboxylation of pyruvate. This means that, while at first the nodule is fixing CO_2_, later is losing it. In this sense, nodule CO_2_ fixation may represent a C-saving mechanism particularly in occasions of limited C availability (Fischinger and Schulze, 2010; Fischinger et al., 2010), such as the ones infected with SFJ14-36 strain. This Asp produced may be exported through xylem or used for glutamic acid formation and consequently the production of glutamine by glutamine synthetase (GS) and ureides (Lima and Sodek, 2003). The high levels of uric acid and allantoin in nodules of plants inoculated with SFJ14-36 strain at a[CO_2_] could support the idea of Asp as an intermediate metabolite in N assimilation under limited C supply to the nodule. However, more studies are necessary to proof this N assimilation route in determinate nodules. These results are in accordance with the ^15^N labeled data (Figures 2, 3A) which showed a good SFJ14-36 nodule performance (measured as nodule δ^15^N) at both CO_2_ levels, similar to the one observed in the reference strain (USDA110) at e[CO_2_], however, due to a deficient nodulation in SFJ14-36 at a[CO_2_] the total amount of N_2_ fixed by nodules was very low (Figure 3A).

On the other hand, the accumulation of ureides, uric acid and allantoin (Serraj et al., 2001; Ladrera et al., 2007), and Asp (King and Purcell, 2005) has been proposed in soybean nodules to take part in the modulation of the symbiotic activity, acting as an N-feedback mechanism. In our study, allantoin content, similar to Asp, was strongly affected by CO_2_ level, especially in plants infected with SFJ14-36 strain, suggesting that the greater content of this N-transporting compound under a[CO_2_] could be related with a decline in shoot N demand (Serraj et al., 1999); however, N-feedback effect by these compounds had not been observed since nodule performance (measured as δ^15^N) was not reduced at a[CO_2_] (Figure 2). Additionally, the levels of organic acids implied in Krebs cycle (malate, fumarate, citrate, α-KG) were similar at those observed in plants with this strain grown at e[CO_2_], suggesting an active nodule N-fixation but a poor N transport to aerial tissues in SFJ14-36 grown at a[CO_2_]. All this data could imply that poor plant fitness observed in SFJ14-36 at a[CO_2_] was at the end caused by a N stress. N deficiency was due to a poor nodule implantation, in terms of biomass, to an insufficient C import from leaves (low levels of sucrose), and to a poor N transport of nodule-fixed N that affects N status at whole plant level reducing C fixation through photosynthesis and so total biomass. Nevertheless, these nodules consumed all the sucrose from the aerial part (Figure 7) and they try to fix N in a more efficient way through the carboxylation of PEP to produce Asp; however, more work is needed to confirm the last hypothesis.

### Photorespiration Enhance at a[CO_2_] Causes a Reorchestration on Krebs Cycle and Glutamic Acid Production in Leaves

Carbon dioxide and O_2_ are competitive substrates for ribulose-l,5-bisphosphate carboxylase/oxygenase (Rubisco), and their ratio at the site of catalysis affects the rates of ribulose-1,5-bisphosphate (RuBP) carboxylation and oxygenation (Farquhar et al., 1980; Rachmilevitch et al., 2004). For this reason, increasing atmospheric [CO_2_] is expected to promote photosynthesis (C reduction) over photorespiration (C oxidation) as showed in C3 plants (Lin and Wang, 2002; Woodward, 2002) and seen here with the RuBP oxygenation (*v*_o_) estimation (Figure 4A). Our results showed higher concentration of leaf metabolites involved in photorespiratory pathway (glycolate, Gly, Ser, and glycerate) in plants infected with SFJ4-24 and SFJ14-36 grown at a[CO_2_], although the difference between CO_2_ levels was greater in SFJ14-36. Additionally, all four metabolites showed a similar trend in the combined response to *Bradyrhizobium* strain and CO_2_ level as shown in the heat map where they were grouped in the same cluster (Figure 6B) indicating a clear relationship between them. These metabolic results were in accordance with the estimation of *v*_o_, where significant reductions in *v*_o_ were found between CO_2_ levels for plants inoculated with SFJ4-24 and SFJ14-36 strains but not for USDA110 (Figure 4A), and previous works with soybeans (Booker et al., 1997; Rogers et al., 2006; Ainsworth and Rogers, 2007).

As in the case of photorespiration, some studies had shown that under a[CO_2_], respiration and therefore Krebs cycle was up-regulated when compared with plants grown at e[CO_2_] (Tcherkez et al., 2008; Soba et al., 2019a, b). Sanz-Sáez et al. (2019) showed that leaves of soybean inoculated with SFJ14-36 showed a significant increase of respiration at a[CO_2_] when compared with e[CO_2_]; meanwhile, plants inoculated with USDA110 and SFJ14-36 did not show respiratory differences between CO_2_ levels. Nevertheless, under a[CO_2_], respiration has been suggested to be inhibited by the high levels of mitochondrial NADH, from photorespiration, and ATP/ADP, from photosynthesis (Padan et al., 2005). On the contrary, as commented before, higher Glu quantities are demanded with increased photorespiration rates and, therefore, an increase in 2-oxoglutarate coming from Krebs cycle is expected. Consequently, a complex respiratory homeostasis between two opposing forces: mitochondrial energy requirements and photorespiratory Glu demand is observed in leaves. Our data showed a general increase of dicarboxylic acids involved in Krebs cycle (fumarate, succinate, citrate, and malate) under a[CO_2_] that was especially marked in the case of SFJ14-36 strain, which is in accordance with previous respiratory data showed by Sanz-Sáez et al. (2019) and with the enhanced Glu demand by photorespiration in SFJ14-36 under a[CO_2_]. Interestingly, α-KG was not significantly affected by CO_2_ treatment in SFJ14-36 and may be due to the fact that is involved in the synthesis of Glu and Gln and, therefore, diverted at a[CO_2_] to the production of these two amino acids.

In summary, the observed enhancement of photorespiration in plants inoculated with SFJ14-36 strain grown at a[CO_2_] alters the leaf respiratory homeostasis between the downregulation caused by more NADH and the upregulation by more Glu demand. Our data suggest that at a[CO_2_], plants inoculated with SFJ14-36 strain showed an upregulation of Krebs cycle to compensate the demand of C skeletons for Glu production. We hypothesize that the imbalance of energy between production (through photosynthesis) and consumption (photorespiration and respiration) observed in SJF14-36 at a[CO_2_] was compensated by a greater fatty acid synthesis as seen in the significant increase of all free fatty acids analyzed (Figures 6B, 8). This could also indirectly reflect a diversion of photoassimilates to the synthesis of organic acids produced by a lower sucrose export to the nodule especially at a[CO_2_].

### Soybean Inoculated With SFJ14-36 Strain Prematurely Induced Leaf Senescence When Grown at a [CO_2_] Probably Caused by N Deficiency

In addition to leaf ontogenic senescence that occurs during the normal aging, prematurely induced senescence can occur when plants are subjected to abiotic/biotic stresses (Troncoso-Ponce et al., 2013). One of the first events that happen is chlorophyll degradation and, as a result, free phytol is produced which can be used as a biomarker of the rapid loss of chlorophyll associated with the degeneration of chloroplast under stress (Lim et al., 2007). In our case, free phytol content in plants inoculated with SFJ14-36 strain at a[CO_2_] was five times greater compared with e[CO_2_] and with the other inoculation treatments (Figure 8), suggesting an additional stress in this treatment, probably due to a poor supply of N by the nodules.

The resulting free phytol is highly toxic to proteins and membranes, so a large proportion of phytol is incorporated into α-tocopherol, fatty acid phytyl esters, and triacylglycerol (Peisker et al., 1989; Ischebeck et al., 2006; Figure 8). α-Tocopherol is the most important lipophilic antioxidant in leaves (Munné-Bosch, 2007), protecting membrane lipids against lipid peroxidation. In our work, α-tocopherol content observed in leaves of SFJ14-36 plants at a[CO_2_] was not significantly higher compared with e[CO_2_] despite the greater phytol content observed at a[CO_2_]. Two possible explanations could be: (1) a poor α-tocopherol synthesis from phytol, in opposition to previous works (Lippold et al., 2012; von Dorp et al., 2015; Mach, 2015) or (2) high tocopherol degradation that provides an excess in their synthesis. The last option could occur when the stress is too severe and consequently lipid peroxidation increases (Munné-Bosch, 2005) and is likely to happen in our case. In addition to α-tocopherol, salicylic acid (SA) increased by 6.3-fold at a[CO_2_] in comparison with e[CO_2_] in SFJ14-36 plants. Several investigations indicated that SA increases accumulation of phenolics (Kováčik et al., 2008) and enhances the oxidative stress tolerance (Li et al., 2014) through stimulation of enzymatic and non-enzymatic antioxidant mechanism pathways (El-Esawi et al., 2017). In accordance to this, phenolic acids such as caffeic acid, ferulic acid, and coumaric acid were also upregulated at a[CO_2_] in SFJ14-36 plants (Figure 6B). Phenolic acids have been reported to be antioxidants that are implied in the scavenging of free radicals (Ghasemzadeh and Ghasemzadeh, 2011). These results indicated that, the antioxidant system was activated due to a severe oxidative stress specifically in leaves of soybeans inoculated with SFJ14-36 strain at a[CO_2_] but not in other treatments.

In plants, increased production of Reactive Oxygen Species and the accumulation of lipid peroxidation products have been associated with oxidation of membrane lipids and membrane catabolism during environmental stresses or senescence (Barclay and McKersie, 1994; Berjak and Pammenter, 2008). The amount of all the free fatty acid analyzed (capric, lauric, palmitic, palmitoleic, oleic, elaidic, arachidic, and linoleic acid) showed a significant increase at a[CO_2_] when compared with e[CO_2_] in plants inoculated with SFJ14-36 strain. However, in the other two strains, the values remained unaltered between CO_2_ levels. During maturation, aging, and senescence, the catabolic enzymatic activities become activated, and free fatty acids have been found to be enhanced considerably (Mishra et al., 2006). Together with free fatty acids, sterols (β-sitosterol and stigmasterol) were enhanced at a[CO_2_] only in SFJ14-36 plants, remaining unchanged in the other treatments. Sterols have also been found to be enhanced in senescing leaves (Duperon et al., 1984; Bouvier-Navé et al., 2010; Li et al., 2016) where they appear to participate in recycling these fatty acids released from senescing cell membranes to form sterol esters for subsequent transport to other tissues (Holmer et al., 1973; Chen et al., 2007).

All these metabolomic data suggest that in leaves of soybeans inoculated with SFJ14-36 strain grown at a[CO_2_], chlorophyll degradation and, therefore, lower photosynthetic ability was likely to occur at the same time as thylakoid membrane degradations. This was previously proposed by Li et al. (2016) during leaf senescence, in our case, probably due to enhancement of oxidative stress and N deficiency. However, this happened only when the plants were grown at current CO_2_ conditions not at e[CO_2_], showing a soybean-strain specific interaction with atmospheric [CO_2_]. As N is essential for synthesis of Rubisco and chlorophylls, in soybean plants inoculated with SFJ14-36 strain, insufficient N-fixation at a[CO_2_] (Figure 3C) decreased leaf N concentration and, as a consequence, photosynthetic rates were decreased in this *B. japonicum* strains (Sanz-Sáez et al., 2019). In this respect, some studies have shown that a sufficient supply of N through BNF increases photosynthetic rates and delays leaf senescence in soybean (Abu-Shakra et al., 1978; Kaschuk et al., 2010). Therefore, observed induced leaf senescence in SFJ14-36 may be due to an insufficient supply of N from the BNF to the leaves as already observed in soybean (Egli et al., 1978). As stated before, the poor supply of N from nodules to leaves was not likely due to low nodule BNF efficiency (Figure 2) but probably to a deficient nodulation as observed by the significant reduction in nodule biomass in SFJ14-36 grown at a[CO_2_] when compared with e[CO_2_].

## Conclusion

In this study, with the use of metabolomics and their interaction with physiological measures in soybean nodules and leaves, we revealed alterations in metabolites response to CO_2_ fertilization in plants inoculated with different *Bradyrhizobium* strains. This analysis clearly demonstrated that the soybeans inoculated with the strain isolated at e[CO_2_] (SFJ14-36) when grown at a[CO_2_] suffers changes in metabolic pathways that affect negatively plant growth and development such as a restricted photoassimilate content (sucrose, glucose, fructose). We hypothesize that under these conditions in the nodule, a more efficient use of C and N happened through a carboxylation of PEP to produce Asp instead of decarboxylation of PEP to produce Krebs dicarboxylic acids. In this way, nodule CO_2_ fixation may represent a C-saving mechanism and Asp can be used as N-exported compound or to produce ureides. At leaf level, plants inoculated with SFJ14-36 and grown at a[CO_2_] showed a complete rearrangement of processes such as photorespiration and Krebs cycle. This metabolic change at a[CO_2_] in plants inoculated with SFJ14-36 that were originally isolated at e[CO_2_], was probably due to a poor nodulation caused by a change in plant root exudates between elevated and ambient [CO_2_], affecting legume-bacteria interaction and, as a result, reducing N_2_-fixation and affecting N metabolism. In parallel, induced senescence likely happened as a result of N deprivation and was shown by the enhanced levels of free phytol, free fatty acids, and other compound related with chlorophyll and membrane degradation that may be caused by an oxidative stress due to the poor N status. The metabolism in leaves of the plants inoculated with the SFJ14-36 strain and grown at a[CO_2_] seems to swift from C assimilation to catabolism of chlorophyll and macromolecules such as fatty acids caused by N deficiency. However, more research is needed with other strains isolated at e[CO_2_] and more soybean cultivars in order to confirm this observations.

## Data Availability Statement

The original contributions presented in the study are included in the article/Supplementary Material, further inquiries can be directed to the corresponding author/s.

## Author Contributions

DS: formal analysis and writing original draft. IA: conceptualization, resource managing, review and editing. UP-L: experimentation, data curation, review, and editing. AM-P: experimentation, data curation, review, and editing. AM-R: resource managing, experimentation, review, and editing. ML: conceptualization, resource managing, experimentation, supervision, review, and editing. AS-S: conceptualization, experimentation, data curation, formal analysis, project administration, supervision, and writing original draft. All authors contributed to the article and approved the submitted version.

## Conflict of Interest

The authors declare that the research was conducted in the absence of any commercial or financial relationships that could be construed as a potential conflict of interest.

## Footnotes

1 www.Co2.earth

2 https://soyface.illinois.edu

## Supplementary Material

Supplementary Figure 1 Classification of metabolites detected by GC-TOF/MS-based analysis in leaves and nodules of plants inoculated with USDA110, SFJ4-24, and SFJ14-36 *B. japonicum* strains under ambient and elevated [CO_2_].
