# Supplementary material for: Soybean Inoculated With One Bradyrhizobium Strain Isolated at Elevated [CO2] Show an Impaired C and N Metabolism When Grown at Ambient [CO2]
Source: Front Plant Sci. 2021 May 20;12:656961. doi: 10.3389/fpls.2021.656961 (PMC8173217; doi:10.3389/fpls.2021.656961)
Supplement: Supplementary Figure 1 — Classification of metabolites detected by GC-TOF/MS-based analysis in leaves and nodules of plants inoculated with USDA110, SFJ4-24, and SFJ14-36 B. japonicum strains under ambient and elevated [CO2]. [file Data_Sheet_1.PDF]

Supplementary Material

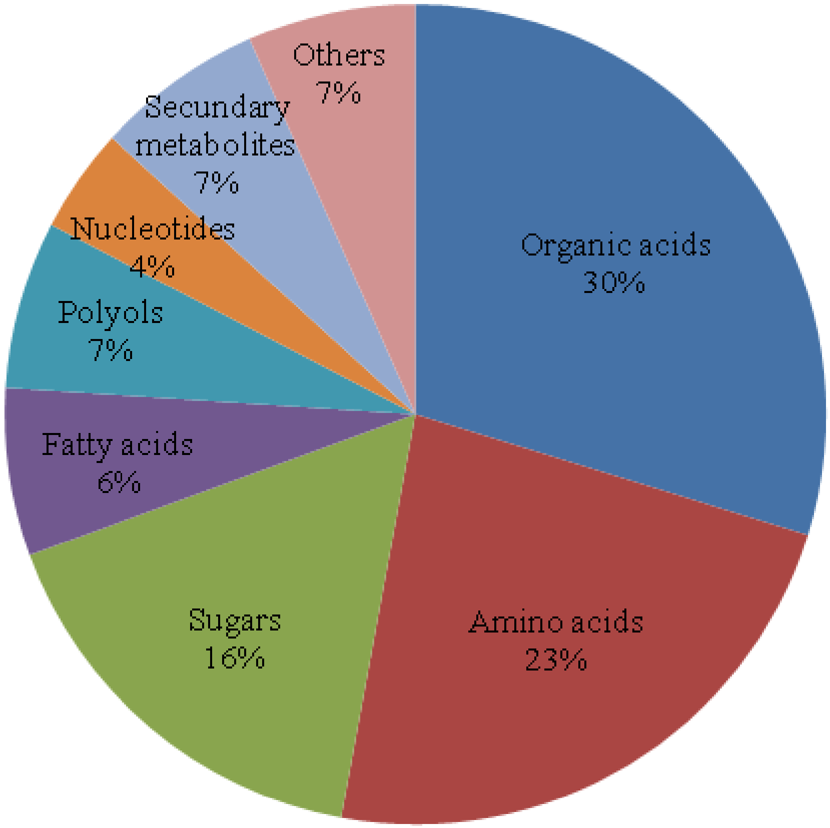

Supplementary Figure 1. . Classification of metabolites detected by GC-TOF/MS-based analysis in leaves and nodules of plants inoculated with USDA110, SFJ4-24 and SFJ14-36 Bradyrhizobium japonicum strains under ambient and elevated [CO2].
